# Moving beyond the search for the first discoverer of autism

**DOI:** 10.3389/fpsyt.2024.1266486

**Published:** 2024-01-17

**Authors:** Marga Vicedo

**Affiliations:** Institute for the History and Philosophy of Science and Technology, University of Toronto, Toronto, ON, Canada

**Keywords:** autism, discovery of autism, Kanner, Asperger, history of autism, simultaneous discovery

## 1 Introduction

In 1968 *Acta Psychopaediatrica*, the first journal of child psychiatry, celebrated the 25th anniversary of the discovery of autism. Its editor, the Dutch child psychiatrist Arnold van Krevelen, called Johns Hopkins University child psychiatrist Leo Kanner and University of Vienna pediatrician Hans Asperger “godfathers” to the first autistic children [(1), p. 97]. Kanner had written in 1943 about “*inborn autistic disturbances of affective contact*” and postulated a “syndrome,” characterized by some children's “*inability to relate themselves* in the ordinary way to people and situations from the beginning of life” [(2), p. 250, 242]. The following year, he named their condition “early infantile autism” (3). In 1944, Asperger also published about children who had various skills and high cognitive capacities but displayed difficulties in relating to others socially. He called their condition “autistic psychopathy,” -psychopathy then being used to design a personality type (4). For van Krevelen the temporal closeness between Kanner's and Asperger's publications was just a coincidence that resulted from the fact that “new discoveries are period-bound rather than area-bound” and they often emerged at the same time in different geographical areas [(5), p. 82].

Recent scholarship, however, has complicated this picture about the discovery of autism. In 2015, journalist Steve Silberman accused Kanner of plagiarizing Asperger and criticized van Krevelen for wrongly promoting the “myth of serendipity” [(6), p. 220]. But just a few years later historians of science Marga Vicedo and Juan Ilerbaig showed that the available historical evidence contradicts Silberman's position (7). In addition, several scholars have published books about different countries which highlight the diversity of actors that have played a role in the history of autism (8–15). Still, several accounts focus on identifying the first discoverer of autism (16–21). Some authors have hailed the Soviet psychiatrist Grunya Efimovna Sukhareva as the first because she published some of the earliest descriptions of children with autistic characteristics (20, 22–26).

But what counts as “discovering autism?” Should the first researcher who used the word “autism” be recognized as its discoverer? Or the first one who described a person with autistic characteristics—whether that scientist used the term “autism” or not? Or the first who postulated autism as a unique syndrome? Or should it be the first one who conceptualized autism as the scientific community does today? There is no agreement on how to answer those questions.

Here, I argue that the search for the first discoverer of autism is not a fruitful way of approaching the history of this condition, which is better understood as a collective effort. In support of this view, this paper makes three claims: (1) autism was not “discovered” by one or even two individuals; (2) the problem of the alleged simultaneous discovery of autism by Asperger and Kanner dissolves when we recognize that numerous people contributed to the early conceptualization of the condition; (3) the search for the first discoverer of autism is misguided because it obscures the communal and historical construction of mental conditions.

## 2 Discovery as a collective effort

In this section, I present a brief look at the early history of autism research to show that the identification of autism as an independent condition was a group effort that took place over several decades.

In the 1910's, the Swiss psychiatrist Eugen Bleuler introduced the term autism to refer to what he identified as one of the symptoms of adult schizophrenia, namely a person's tendency to turn away from reality and retire into a subjective world. For Bleuler, this autistic behavior was deeply connected to the person's affective life (27). Though Bleuler also talked about “autistic thinking” as a type of thinking disconnected from logic and reality that all individuals engaged in occasionally (28), psychiatrists focused on examining autistic behavior as a key element in adult schizophrenia (29–31).

Around the mid-1920's, as the nascent field of child psychiatry started to bloom, the study of autistic behavior in children diagnosed as schizophrenic revealed that they displayed a wide range of behaviors and different life histories. German psychiatrist Fritz Künkel examined the childhood of more than 100 schizophrenic patients as reported in their clinical histories. He divided them into four groups with distinctive complexes of symptoms, which he called pedantic, asocial, irritable, and, autistic. In the autistic group, Künkel emphasized disturbances “in the field of affectivity” [(32), p. 269]. Citing Bleuler's and Künkel's work, Sukhareva (Ssucharewa in the German publications cited here) published a 1926 article describing six boys who were musically gifted and with a tendency toward abstract, orderly, and precise thinking. She labeled their affectivity as “flattened,” and noted their “autistic attitude” because the boys avoided other children. Sukhareva noted that the children had shown progress since her observations began. For that reason, she argued that the diagnosis of schizophrenia did not seem adequate, as it commonly implied a tendency toward disintegration of the personality. Instead, she claimed to be describing “schizoid psychopathies” in children (33), and continued to publish about other similar cases (34, 35).

During the 1930's, numerous European and North American psychiatrists and pediatricians studied autistic behavior in children, often focusing on its relation to their social and affective relations. Sukhareva reported on a larger group of children who had difficulties in the “affective contact with their surroundings,” developed unusual interests such as calculations and astronomical studies, and displayed “lack of adaptability” and “autism” (Ssucharewa (36), p. 312). Grebelskaja-Albatz (37) described children who showed an “alteration of the affective life” and autism. In Vienna, Georg Frankl described some children who exhibited difficulties in social relations because of their inability to understand the affective tone of language. For Frankl, they were affected by a “disturbance of emotional contact,” an interruption of the affective contact that led them to a total absence of relationships with people in their environment: an extreme autism (38).

This short presentation of some contributions to the early history of autism shows that Kanner and Asperger were not the first to use the term autism nor the first to diagnose children with autism in the sense of self-isolating behavior. Neither were they the first to note the great diversity among these children and the fact that many of them did not fit well under the category of schizophrenia.

Yet, in the early 1940's Kanner and Asperger put forward autism as a new syndrome, different from childhood schizophrenia. Kanner postulated “infantile autism” (2, 3) as a condition of the affects, one characterized by the children's extreme aloneness, speech disturbances (echolalia, repetition of phrases), and obsessive desire for sameness. Though he had introduced the category of “autistic psychopathy” in 1938, Asperger's best known 1944 paper presented this condition as a diagnosis for children with some special skills and high intelligence who also had narrow interests, a tendency for self-isolation, and difficulties in social relations (4, 39).

## 3 Simultaneous discovery as a misleading retrospective assessment

Was Kanner's and Asperger's presentation of autism as a unique syndrome a case of simultaneous discovery? Since Kanner and Asperger were not the first to describe autistic children or to use the term autism, the question can only be whether they conceptualized the condition of autism in the same manner. The answer is that they did not because they came from different research traditions. Asperger adopted a typological framework that aimed to identify types of individuals with specific mental characteristics, very different from Kanner's emphasis on the unique development of each individual. As a result of their diverse approaches and their observations of children with different degrees of autistic behavior and cognitive capacities, Kanner's and Asperger's conceptions of autism were more different than has so far been appreciated. Moreover, Kanner and Asperger themselves maintained that they had identified different conditions (40), but many scientists and historians have paid little heed to their views on this issue. The focus on establishing priority in the “discovery” of autism has obscured this important aspect of the history of medical views about the condition.

Many alleged similarities between Kanner's and Asperger's syndromes have resulted from a de-contextualized understandings of their frameworks for mental conditions and from attributing modern meanings to some concepts they used. As historians of physics Thomas Kuhn and Peter Galison found in their work on simultaneous discoveries in physics, the perception of simultaneity is often a retrospective assessment that results from the imposition of current views about a phenomenon on earlier accounts (41–43). In fact, some of the similarities between Kanner's and Asperger's ideas were “created” by later researchers. For example, when Uta Frith translated Asperger's 1944 paper from German into English, she omitted the preface where he talked about psychopathy, and translated some terms into English with a contemporary meaning. She thus made Asperger sound more “Kannerian” than he was (44).

But some of the similarities between Kanner's and Asperger's views should not be surprising given that many researchers had already discussed several aspects of autism. Understanding better the relationships among them would likely dissolve the puzzle of simultaneity. If many scientists are working on a particular problem at a given time, it does not seem puzzling that some of them would arrive at ideas that are similar in some respects.

## 4 Why searching for the first discoverer is not fruitful

It would be better to abandon the search for the first discoverer of autism because it is at odds with our understanding of scientific development as a communal endeavor and it also conceals the historical nature of mental conditions.

The search for the “first discoverer” of autism contributes to a perception of science as an individualistic pursuit, an enterprise led by a handful of extraordinary minds often ahead of their time, rather than a complex collective process. However, a brief look at the early history of autism research showed that we cannot really identify one or two individuals who “discovered” autism. Numerous investigators published about children with autistic behaviors in the early twentieth century. They provided various insights that led to recognizing that these children did not fit the existing diagnostic categories, such as childhood schizophrenia. Eventually, Kanner and Asperger proposed syndromes with a set of symptoms that have become central in current research on autism.

In addition, the conception of “discovery” as a momentous affair is not fruitful in psychiatry because mental conditions are not “discovered” at a particular point, but are introduced and accepted as a result of a complex interaction between empirical and social factors. Although the analysis of the socio-cultural factors that shaped changing views about autism is beyond the scope of this paper, it is important to note that the focus on the “moment” of discovery neglects the fact that mental conditions are complex hybrid entities that are constructed over time. This does not mean that they are not real; but it does mean that their meaning and significance is the result of a process in which changing cultural beliefs about what a society considers “normal” behaviors influence medical and psychological ideas about a condition and the other way around. From Bleuler's views to contemporary ideas on autism, the conception of autism has been in constant flux as our views about the human mind and “normality” have also evolved over time.

## 5 Conclusion

By focusing on who “discovered” autism first, we have missed the collective effort to conceptualize this condition over the years. No single individual discovered autism in the sense of identifying the condition we label today as such. Mental conditions including autism are constructed through a complex historical process in which different scientific and social actors characterize certain behaviors and modes of thinking as deserving a particular label. Many individuals contribute to this process of meaning-making.

The focus on specific moments of discovery and the search for the discoverer of a mental condition contributes to a view of science that historians have shown to be deeply flawed. Though it is important to examine the role of early pioneers in a field and give credit to those who made valuable contributions, this endeavor should avoid oversimplifying the complex ways in which scientific and social understanding of mental conditions evolve over time.

I propose that a better strategy for grasping the historical nature of mental conditions is to examine the various ways in which different actors (clinicians, researchers, and people diagnosed with a condition and their families) have contributed empirical, conceptual, and experiential knowledge that helped form what eventually was recognized as a unique condition. This historical insight should be valuable to current practitioners by encouraging them to interact with those who can offer different but complementary perspectives and knowledge about a condition.

## Author contributions

MV: Writing – original draft.
